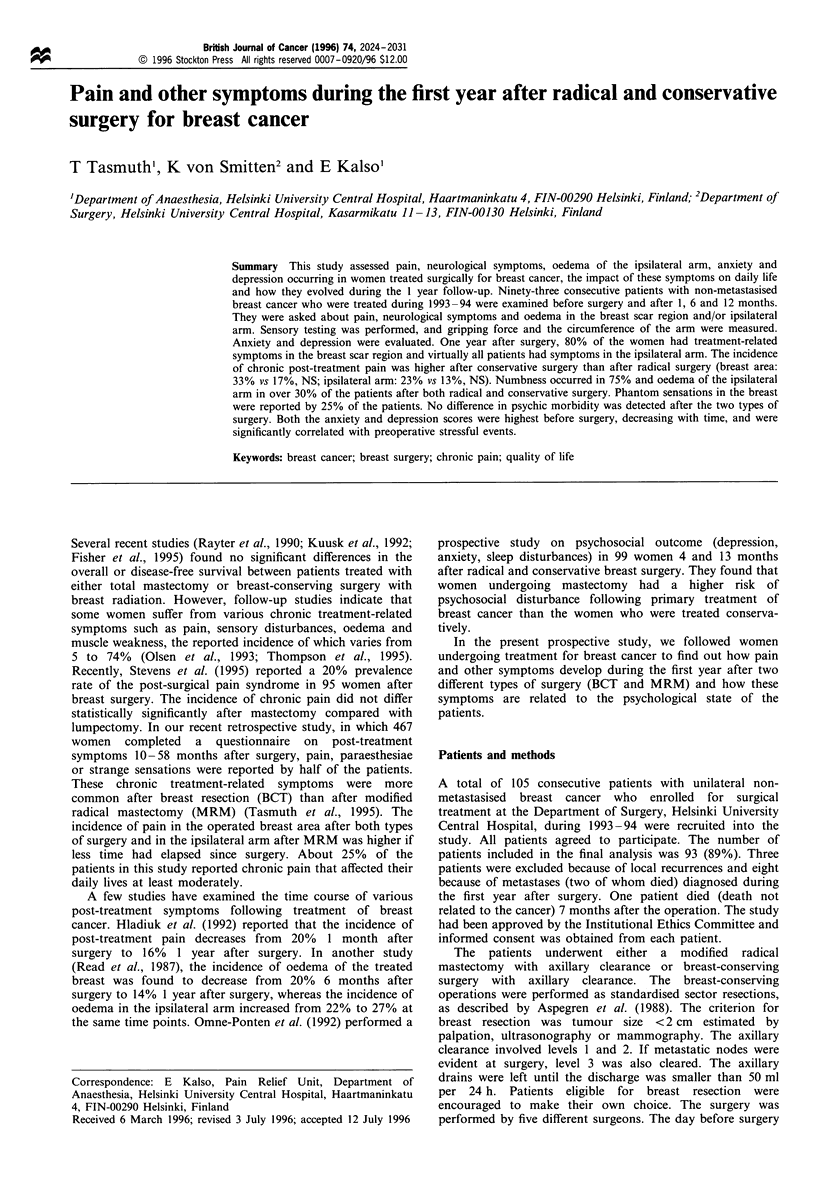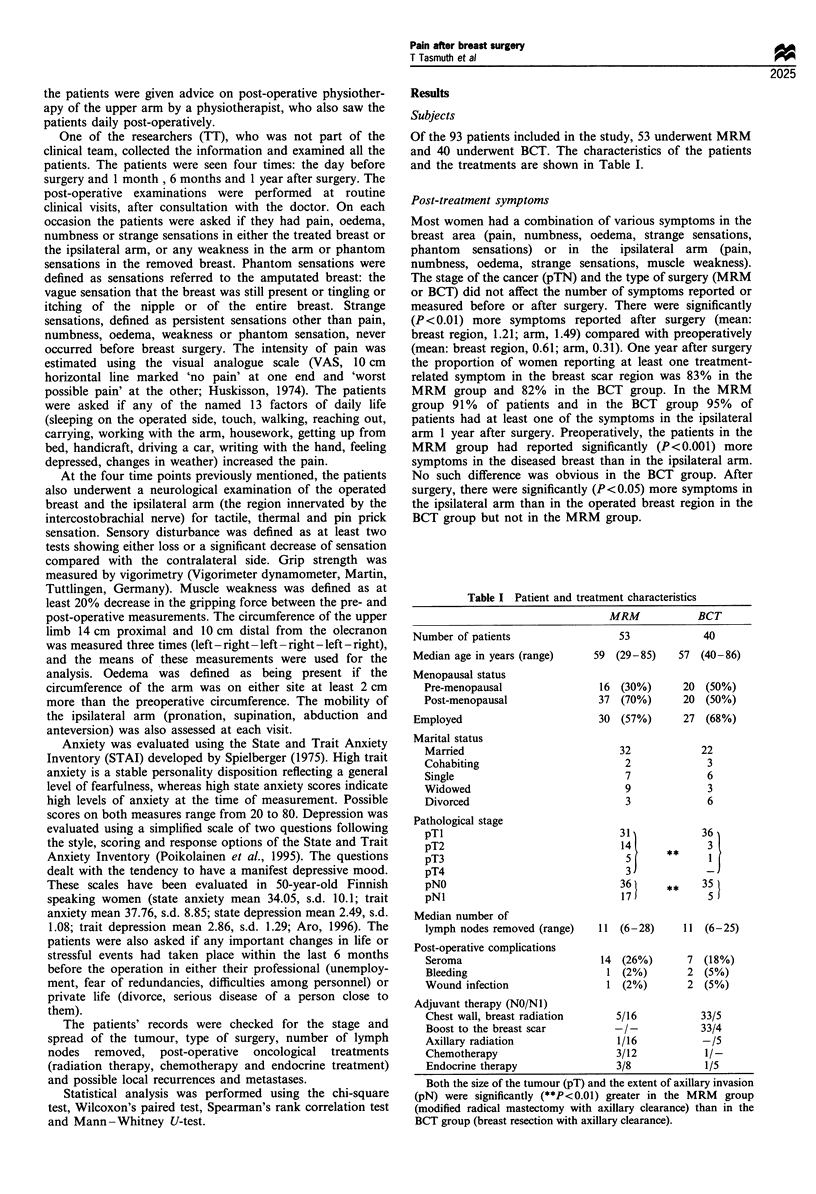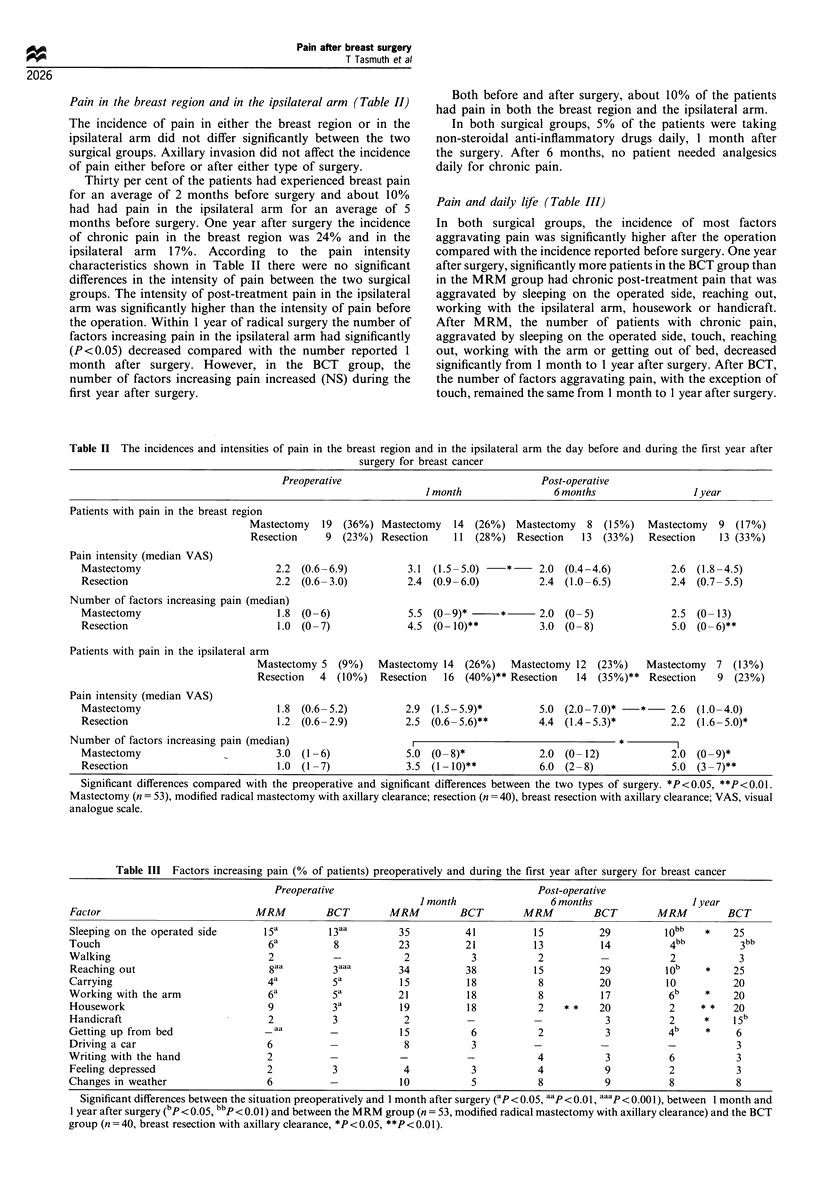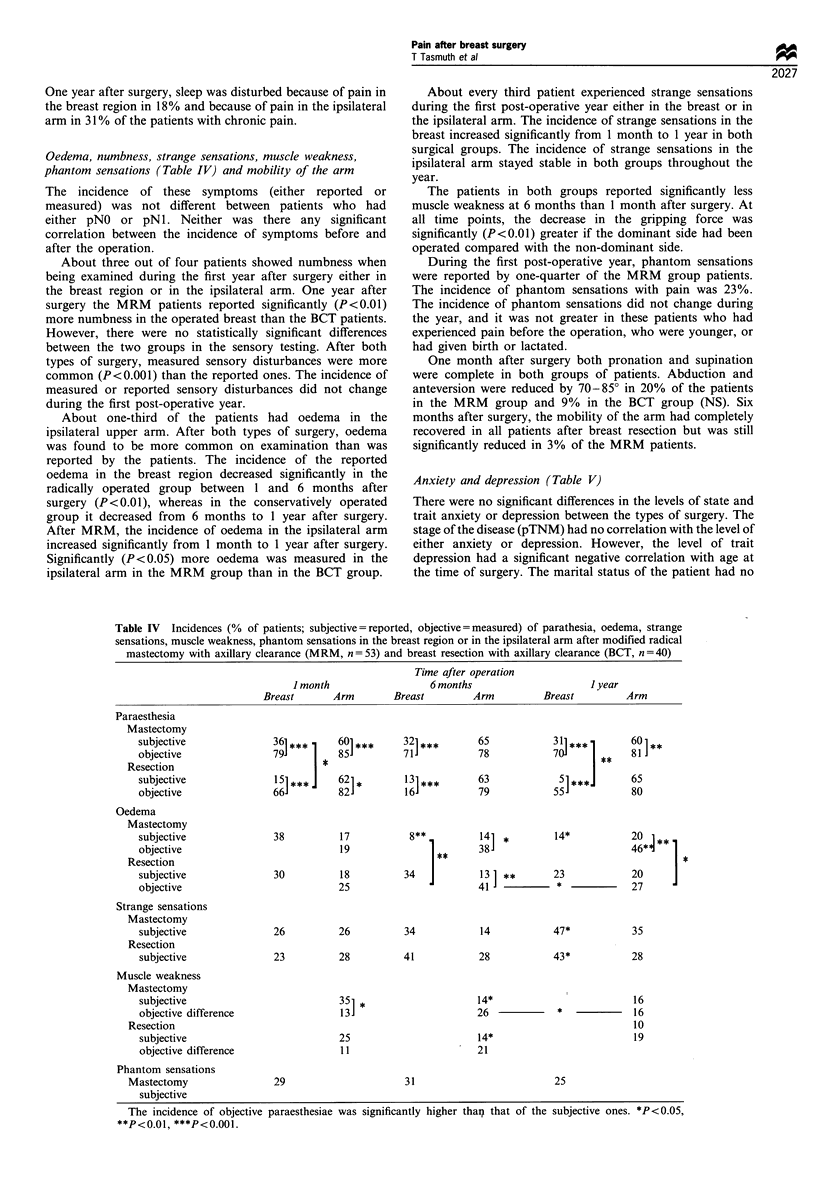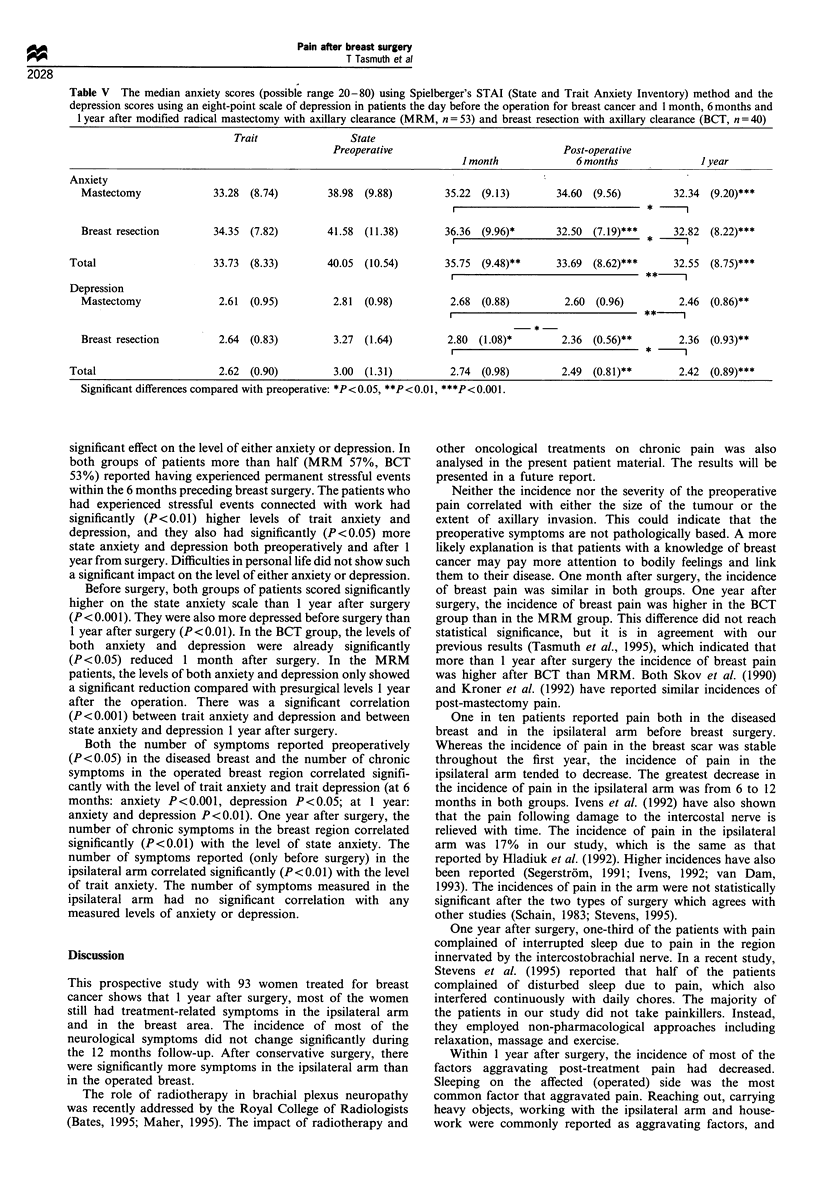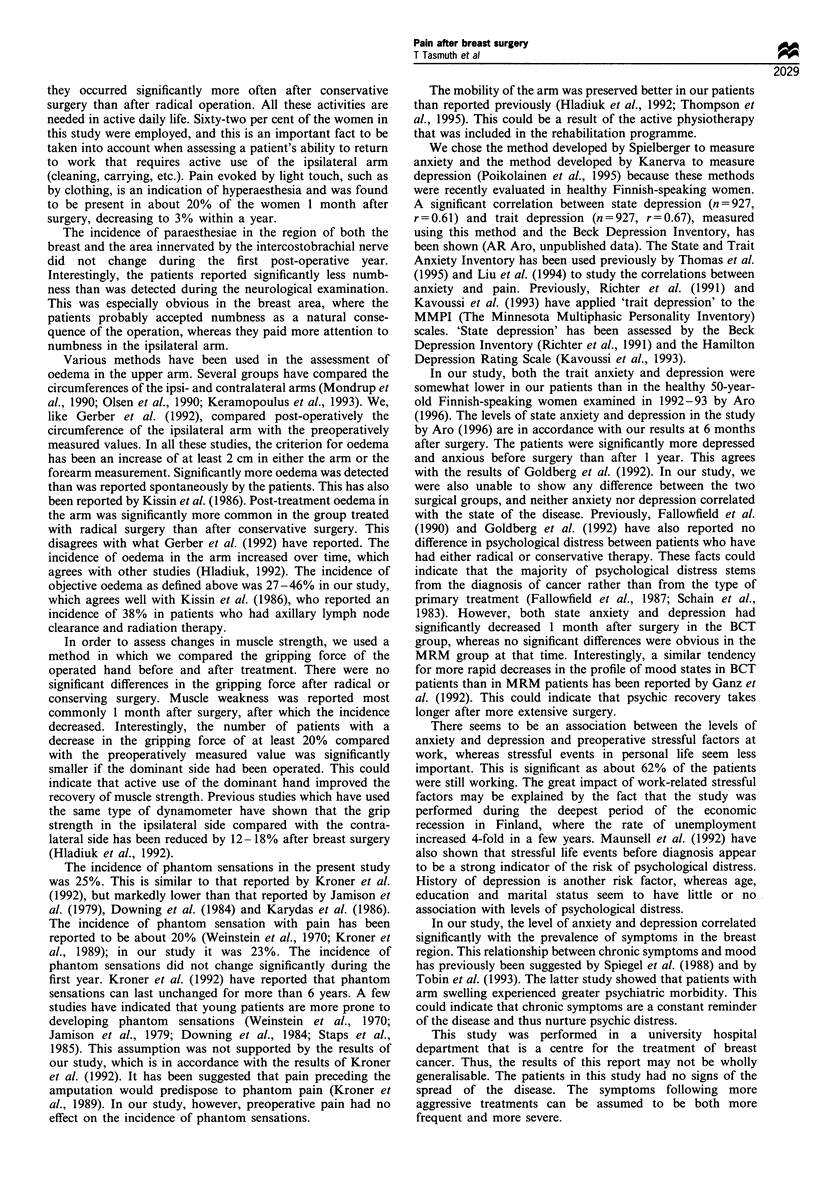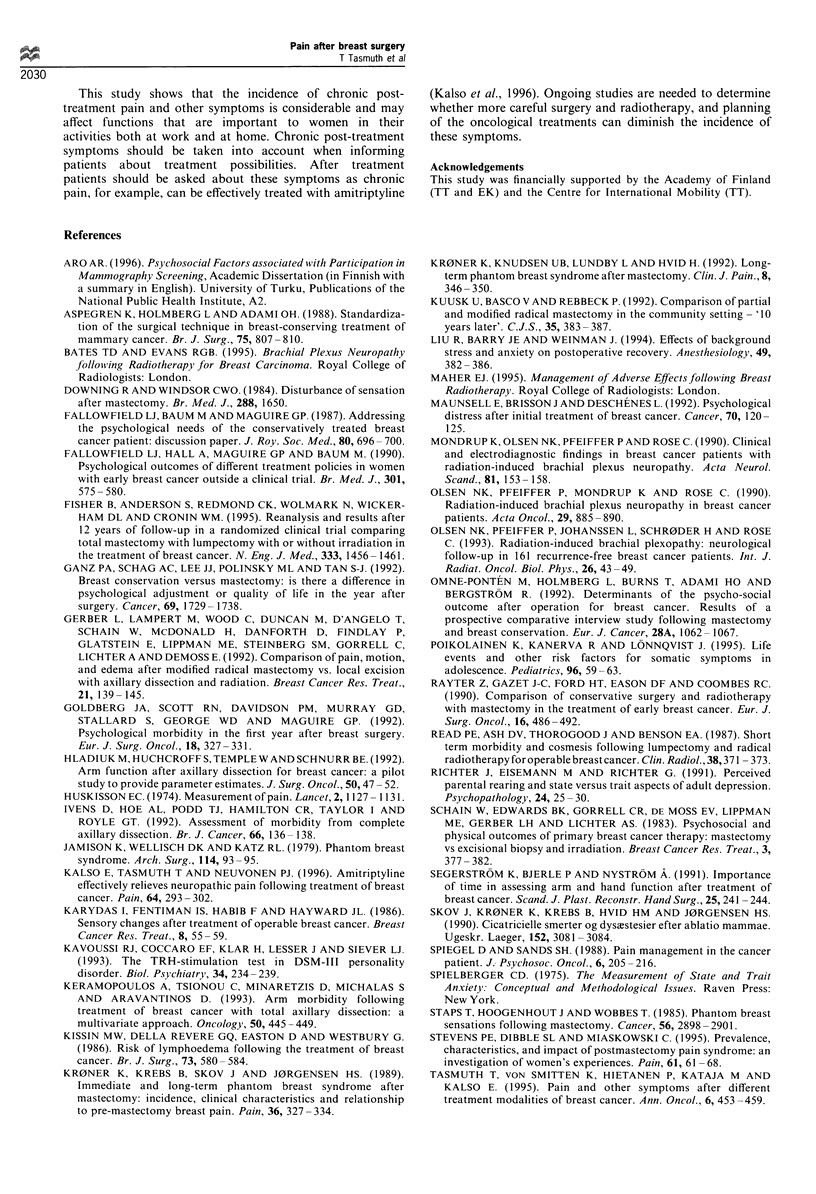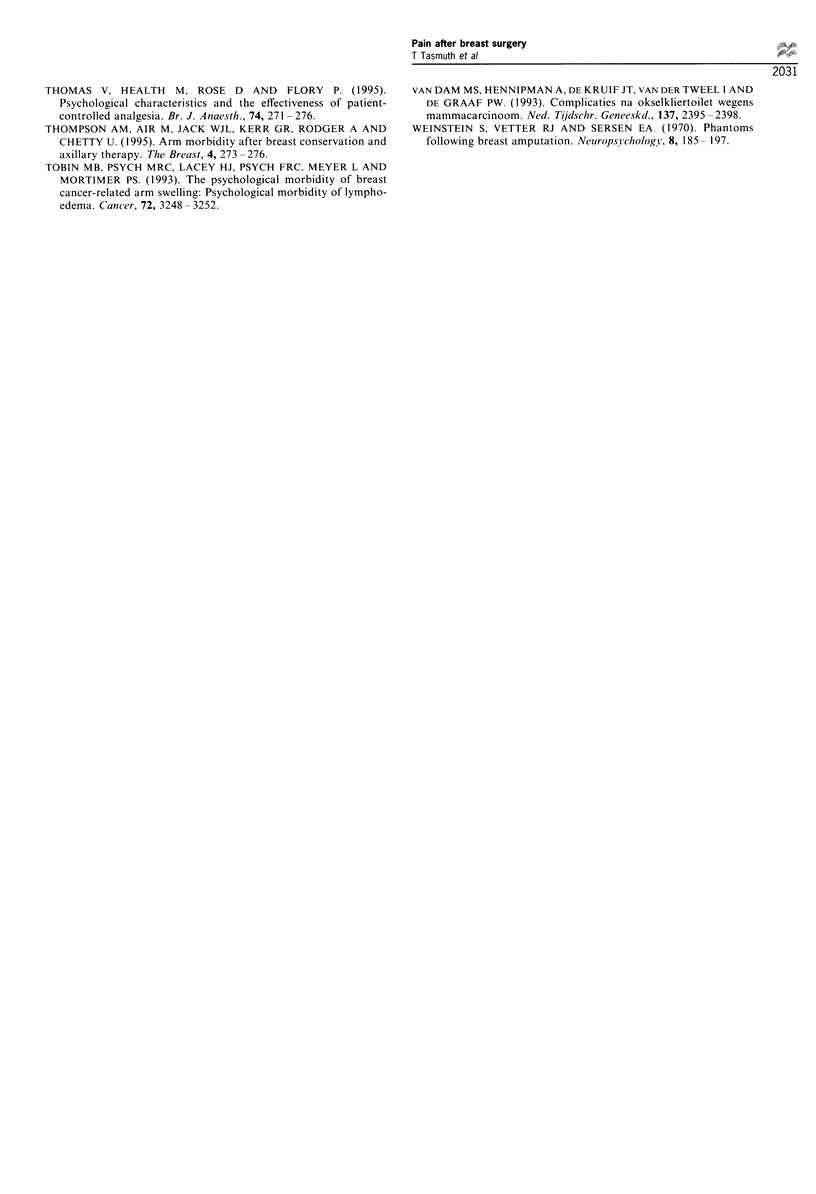# Pain and other symptoms during the first year after radical and conservative surgery for breast cancer.

**DOI:** 10.1038/bjc.1996.671

**Published:** 1996-12

**Authors:** T. Tasmuth, K. von Smitten, E. Kalso

**Affiliations:** Department of Anaesthesia, Helsinki University Central Hospital, Finland.

## Abstract

This study assessed pain, neurological symptoms, oedema of the ipsilateral arm, anxiety and depression occurring in women treated surgically for breast cancer, the impact of these symptoms on daily life and how they evolved during the 1 year follow-up. Ninety-three consecutive patients with non-metastasised breast cancer who were treated during 1993-94 were examined before surgery and after 1, 6 and 12 months. They were asked about pain, neurological symptoms and oedema in the breast scar region and/or ipsilateral arm. Sensory testing was performed, and gripping force and the circumference of the arm were measured. Anxiety and depression were evaluated. One year after surgery, 80% of the women had treatment-related symptoms in the breast scar region and virtually all patients had symptoms in the ipsilateral arm. The incidence of chronic post-treatment pain was higher after conservative surgery than after radical surgery (breast area: 33% vs 17%, NS; ipsilateral arm: 23% vs 13%, NS). Numbness occurred in 75% and oedema of the ipsilateral arm in over 30% of the patients after both radical and conservative surgery. Phantom sensations in the breast were reported by 25% of the patients. No difference in psychic morbidity was detected after the two types of surgery. Both the anxiety and depression scores were highest before surgery, decreasing with time, and were significantly correlated with preoperative stressful events.


British Journal of Cancer (1996) 74, 2024-2031
© 1996 Stockton Press All rights reserved 0007-0920/96 $12.00

### Pain and other symptoms during the first year after radical and conservative surgery for breast cancer

T Tasmuth[1], K von Smitten[2] and E Kalso[1]

[1]*Department of Anaesthesia, Helsinki University Central Hospital, Haartmaninkatu 4, FIN-00290 Helsinki, Finland;* [2]*Department of Surgery, Helsinki University Central Hospital, Kasarmikatu 11-13, FIN-00130 Helsinki, Finland*

**Summary** This study assessed pain, neurological symptoms, oedema of the ipsilateral arm, anxiety and depression occurring in women treated surgically for breast cancer, the impact of these symptoms on daily life and how they evolved during the 1 year follow-up. Ninety-three consecutive patients with non-metastasised breast cancer who were treated during 1993-94 were examined before surgery and after 1, 6 and 12 months. They were asked about pain, neurological symptoms and oedema in the breast scar region and/or ipsilateral arm. Sensory testing was performed, and gripping force and the circumference of the arm were measured. Anxiety and depression were evaluated. One year after surgery, 80% of the women had treatment-related symptoms in the breast scar region and virtually all patients had symptoms in the ipsilateral arm. The incidence of chronic post-treatment pain was higher after conservative surgery than after radical surgery (breast area: 33% *vs* 17%, NS; ipsilateral arm: 23% *vs* 13%, NS). Numbness occurred in 75% and oedema of the ipsilateral arm in over 30% of the patients after both radical and conservative surgery. Phantom sensations in the breast were reported by 25% of the patients. No difference in psychic morbidity was detected after the two types of surgery. Both the anxiety and depression scores were highest before surgery, decreasing with time, and were significantly correlated with preoperative stressful events.

**Keywords:** breast cancer; breast surgery; chronic pain; quality of life

Several recent studies (Rayter *et al.*, 1990; Kuusk *et al.*, 1992; Fisher *et al.*, 1995) found no significant differences in the overall or disease-free survival between patients treated with either total mastectomy or breast-conserving surgery with breast radiation. However, follow-up studies indicate that some women suffer from various chronic treatment-related symptoms such as pain, sensory disturbances, oedema and muscle weakness, the reported incidence of which varies from 5 to 74% (Olsen *et al.*, 1993; Thompson *et al.*, 1995). Recently, Stevens *et al.* (1995) reported a 20% prevalence rate of the post-surgical pain syndrome in 95 women after breast surgery. The incidence of chronic pain did not differ statistically significantly after mastectomy compared with lumpectomy. In our recent retrospective study, in which 467 women completed a questionnaire on post-treatment symptoms 10-58 months after surgery, pain, paraesthesiae or strange sensations were reported by half of the patients. These chronic treatment-related symptoms were more common after breast resection (BCT) than after modified radical mastectomy (MRM) (Tasmuth *et al.*, 1995). The incidence of pain in the operated breast area after both types of surgery and in the ipsilateral arm after MRM was higher if less time had elapsed since surgery. About 25% of the patients in this study reported chronic pain that affected their daily lives at least moderately.

A few studies have examined the time course of various post-treatment symptoms following treatment of breast cancer. Hladiuk *et al.* (1992) reported that the incidence of post-treatment pain decreases from 20% 1 month after surgery to 16% 1 year after surgery. In another study (Read *et al.*, 1987), the incidence of oedema of the treated breast was found to decrease from 20% 6 months after surgery to 14% 1 year after surgery, whereas the incidence of oedema in the ipsilateral arm increased from 22% to 27% at the same time points. Omne-Ponten *et al.* (1992) performed a prospective study on psychosocial outcome (depression, anxiety, sleep disturbances) in 99 women 4 and 13 months after radical and conservative breast surgery. They found that women undergoing mastectomy had a higher risk of psychosocial disturbance following primary treatment of breast cancer than the women who were treated conservatively.

In the present prospective study, we followed women undergoing treatment for breast cancer to find out how pain and other symptoms develop during the first year after two different types of surgery (BCT and MRM) and how these symptoms are related to the psychological state of the patients.

### Patients and methods

A total of 105 consecutive patients with unilateral non-metastasised breast cancer who enrolled for surgical treatment at the Department of Surgery, Helsinki University Central Hospital, during 1993-94 were recruited into the study. All patients agreed to participate. The number of patients included in the final analysis was 93 (89%). Three patients were excluded because of local recurrences and eight because of metastases (two of whom died) diagnosed during the first year after surgery. One patient died (death not related to the cancer) 7 months after the operation. The study had been approved by the Institutional Ethics Committee and informed consent was obtained from each patient.

The patients underwent either a modified radical mastectomy with axillary clearance or breast-conserving surgery with axillary clearance. The breast-conserving operations were performed as standardised sector resections, as described by Aspegren *et al.* (1988). The criterion for breast resection was tumour size <2 cm estimated by palpation, ultrasonography or mammography. The axillary clearance involved levels 1 and 2. If metastatic nodes were evident at surgery, level 3 was also cleared. The axillary drains were left until the discharge was smaller than 50 ml per 24 h. Patients eligible for breast resection were encouraged to make their own choice. The surgery was performed by five different surgeons. The day before surgery

Correspondence: E Kalso, Pain Relief Unit, Department of Anaesthesia, Helsinki University Central Hospital, Haartmaninkatu 4, FIN-00290 Helsinki, Finland
Received 6 March 1996; revised 3 July 1996; accepted 12 July 1996

the patients were given advice on post-operative physiotherapy of the upper arm by a physiotherapist, who also saw the patients daily post-operatively.

One of the researchers (TT), who was not part of the clinical team, collected the information and examined all the patients. The patients were seen four times: the day before surgery and 1 month , 6 months and 1 year after surgery. The post-operative examinations were performed at routine clinical visits, after consultation with the doctor. On each occasion the patients were asked if they had pain, oedema, numbness or strange sensations in either the treated breast or the ipsilateral arm, or any weakness in the arm or phantom sensations in the removed breast. Phantom sensations were defined as sensations referred to the amputated breast: the vague sensation that the breast was still present or tingling or itching of the nipple or of the entire breast. Strange sensations, defined as persistent sensations other than pain, numbness, oedema, weakness or phantom sensation, never occurred before breast surgery. The intensity of pain was estimated using the visual analogue scale (VAS, 10 cm horizontal line marked 'no pain' at one end and 'worst possible pain' at the other; Huskisson, 1974). The patients were asked if any of the named 13 factors of daily life (sleeping on the operated side, touch, walking, reaching out, carrying, working with the arm, housework, getting up from bed, handicraft, driving a car, writing with the hand, feeling depressed, changes in weather) increased the pain.

At the four time points previously mentioned, the patients also underwent a neurological examination of the operated breast and the ipsilateral arm (the region innervated by the intercostobrachial nerve) for tactile, thermal and pin prick sensation. Sensory disturbance was defined as at least two tests showing either loss or a significant decrease of sensation compared with the contralateral side. Grip strength was measured by vigorimetry (Vigorimeter dynamometer, Martin, Tuttlingen, Germany). Muscle weakness was defined as at least 20% decrease in the gripping force between the pre- and post-operative measurements. The circumference of the upper limb 14 cm proximal and 10 cm distal from the olecranon was measured three times (left–right–left–right–left–right), and the means of these measurements were used for the analysis. Oedema was defined as being present if the circumference of the arm was on either site at least 2 cm more than the preoperative circumference. The mobility of the ipsilateral arm (pronation, supination, abduction and anteversion) was also assessed at each visit.

Anxiety was evaluated using the State and Trait Anxiety Inventory (STAI) developed by Spielberger (1975). High trait anxiety is a stable personality disposition reflecting a general level of fearfulness, whereas high state anxiety scores indicate high levels of anxiety at the time of measurement. Possible scores on both measures range from 20 to 80. Depression was evaluated using a simplified scale of two questions following the style, scoring and response options of the State and Trait Anxiety Inventory (Poikolainen *et al.*, 1995). The questions dealt with the tendency to have a manifest depressive mood. These scales have been evaluated in 50-year-old Finnish speaking women (state anxiety mean 34.05, s.d. 10.1; trait anxiety mean 37.76, s.d. 8.85; state depression mean 2.49, s.d. 1.08; trait depression mean 2.86, s.d. 1.29; Aro, 1996). The patients were also asked if any important changes in life or stressful events had taken place within the last 6 months before the operation in either their professional (unemployment, fear of redundancies, difficulties among personnel) or private life (divorce, serious disease of a person close to them).

The patients' records were checked for the stage and spread of the tumour, type of surgery, number of lymph nodes removed, post-operative oncological treatments (radiation therapy, chemotherapy and endocrine treatment) and possible local recurrences and metastases.

Statistical analysis was performed using the chi-square test, Wilcoxon's paired test, Spearman's rank correlation test and Mann–Whitney *U*-test.

### Results

### *Subjects*

Of the 93 patients included in the study, 53 underwent MRM and 40 underwent BCT. The characteristics of the patients and the treatments are shown in Table I.

### *Post-treatment symptoms*

Most women had a combination of various symptoms in the breast area (pain, numbness, oedema, strange sensations, phantom sensations) or in the ipsilateral arm (pain, numbness, oedema, strange sensations, muscle weakness). The stage of the cancer (pTN) and the type of surgery (MRM or BCT) did not affect the number of symptoms reported or measured before or after surgery. There were significantly ($P<0.01$) more symptoms reported after surgery (mean: breast region, 1.21; arm, 1.49) compared with preoperatively (mean: breast region, 0.61; arm, 0.31). One year after surgery the proportion of women reporting at least one treatment-related symptom in the breast scar region was 83% in the MRM group and 82% in the BCT group. In the MRM group 91% of patients and in the BCT group 95% of patients had at least one of the symptoms in the ipsilateral arm 1 year after surgery. Preoperatively, the patients in the MRM group had reported significantly ($P<0.001$) more symptoms in the diseased breast than in the ipsilateral arm. No such difference was obvious in the BCT group. After surgery, there were significantly ($P<0.05$) more symptoms in the ipsilateral arm than in the operated breast region in the BCT group but not in the MRM group.

**Table I** Patient and treatment characteristics

| | *MRM* | | *BCT* |
|---|---|---|---|
| Number of patients | 53 | | 40 |
| Median age in years (range) | 59 (29–85) | | 57 (40–86) |
| Menopausal status | | | |
| Pre-menopausal | 16 (30%) | | 20 (50%) |
| Post-menopausal | 37 (70%) | | 20 (50%) |
| Employed | 30 (57%) | | 27 (68%) |
| Marital status | | | |
| Married | 32 | | 22 |
| Cohabiting | 2 | | 3 |
| Single | 7 | | 6 |
| Widowed | 9 | | 3 |
| Divorced | 3 | | 6 |
| Pathological stage | | | |
| pT1 | 31 | ** | 36 |
| pT2 | 14 | | 3 |
| pT3 | 5 | | 1 |
| pT4 | 3 | | – |
| pN0 | 36 | ** | 35 |
| pN1 | 17 | | 5 |
| Median number of lymph nodes removed (range) | 11 (6–28) | | 11 (6–25) |
| Post-operative complications | | | |
| Seroma | 14 (26%) | | 7 (18%) |
| Bleeding | 1 (2%) | | 2 (5%) |
| Wound infection | 1 (2%) | | 2 (5%) |
| Adjuvant therapy (N0/N1) | | | |
| Chest wall, breast radiation | 5/16 | | 33/5 |
| Boost to the breast scar | –/– | | 33/4 |
| Axillary radiation | 1/16 | | –/5 |
| Chemotherapy | 3/12 | | 1/– |
| Endocrine therapy | 3/8 | | 1/5 |

Both the size of the tumour (pT) and the extent of axillary invasion (pN) were significantly (**$P<0.01$) greater in the MRM group (modified radical mastectomy with axillary clearance) than in the BCT group (breast resection with axillary clearance).

*Pain in the breast region and in the ipsilateral arm (Table II)*

The incidence of pain in either the breast region or in the ipsilateral arm did not differ significantly between the two surgical groups. Axillary invasion did not affect the incidence of pain either before or after either type of surgery.

Thirty per cent of the patients had experienced breast pain for an average of 2 months before surgery and about 10% had had pain in the ipsilateral arm for an average of 5 months before surgery. One year after surgery the incidence of chronic pain in the breast region was 24% and in the ipsilateral arm 17%. According to the pain intensity characteristics shown in Table II there were no significant differences in the intensity of pain between the two surgical groups. The intensity of post-treatment pain in the ipsilateral arm was significantly higher than the intensity of pain before the operation. Within 1 year of radical surgery the number of factors increasing pain in the ipsilateral arm had significantly ($P<0.05$) decreased compared with the number reported 1 month after surgery. However, in the BCT group, the number of factors increasing pain increased (NS) during the first year after surgery.

Both before and after surgery, about 10% of the patients had pain in both the breast region and the ipsilateral arm.

In both surgical groups, 5% of the patients were taking non-steroidal anti-inflammatory drugs daily, 1 month after the surgery. After 6 months, no patient needed analgesics daily for chronic pain.

*Pain and daily life (Table III)*

In both surgical groups, the incidence of most factors aggravating pain was significantly higher after the operation compared with the incidence reported before surgery. One year after surgery, significantly more patients in the BCT group than in the MRM group had chronic post-treatment pain that was aggravated by sleeping on the operated side, reaching out, working with the ipsilateral arm, housework or handicraft. After MRM, the number of patients with chronic pain, aggravated by sleeping on the operated side, touch, reaching out, working with the arm or getting out of bed, decreased significantly from 1 month to 1 year after surgery. After BCT, the number of factors aggravating pain, with the exception of touch, remained the same from 1 month to 1 year after surgery.

**Table II** The incidences and intensities of pain in the breast region and in the ipsilateral arm the day before and during the first year after surgery for breast cancer

| | *Preoperative* | *Post-operative* *1 month* | *6 months* | *1 year* |
|---|---|---|---|---|
| Patients with pain in the breast region | | | | |
| | Mastectomy 19 (36%) | Mastectomy 14 (26%) | Mastectomy 8 (15%) | Mastectomy 9 (17%) |
| | Resection 9 (23%) | Resection 11 (28%) | Resection 13 (33%) | Resection 13 (33%) |
| Pain intensity (median VAS) | | | | |
| Mastectomy | 2.2 (0.6–6.9) | 3.1 (1.5–5.0) —*— | 2.0 (0.4–4.6) | 2.6 (1.8–4.5) |
| Resection | 2.2 (0.6–3.0) | 2.4 (0.9–6.0) | 2.4 (1.0–6.5) | 2.4 (0.7–5.5) |
| Number of factors increasing pain (median) | | | | |
| Mastectomy | 1.8 (0–6) | 5.5 (0–9)* —*— | 2.0 (0–5) | 2.5 (0–13) |
| Resection | 1.0 (0–7) | 4.5 (0–10)** | 3.0 (0–8) | 5.0 (0–6)** |
| Patients with pain in the ipsilateral arm | | | | |
| | Mastectomy 5 (9%) | Mastectomy 14 (26%) | Mastectomy 12 (23%) | Mastectomy 7 (13%) |
| | Resection 4 (10%) | Resection 16 (40%)** | Resection 14 (35%)** | Resection 9 (23%) |
| Pain intensity (median VAS) | | | | |
| Mastectomy | 1.8 (0.6–5.2) | 2.9 (1.5–5.9)* | 5.0 (2.0–7.0)* —*— | 2.6 (1.0–4.0) |
| Resection | 1.2 (0.6–2.9) | 2.5 (0.6–5.6)** | 4.4 (1.4–5.3)* | 2.2 (1.6–5.0)* |
| Number of factors increasing pain (median) | | ┌──── | ──── * ──── | ────┐ |
| Mastectomy | 3.0 (1–6) | 5.0 (0–8)* | 2.0 (0–12) | 2.0 (0–9)* |
| Resection | 1.0 (1–7) | 3.5 (1–10)** | 6.0 (2–8) | 5.0 (3–7)** |

Significant differences compared with the preoperative and significant differences between the two types of surgery. $*P<0.05$, $**P<0.01$. Mastectomy ($n=53$), modified radical mastectomy with axillary clearance; resection ($n=40$), breast resection with axillary clearance; VAS, visual analogue scale.

**Table III** Factors increasing pain (% of patients) preoperatively and during the first year after surgery for breast cancer

| Factor | *Preoperative* MRM | BCT | *Post-operative* *1 month* MRM | BCT | *6 months* MRM | | BCT | *1 year* MRM | | BCT |
|---|---|---|---|---|---|---|---|---|---|---|
| Sleeping on the operated side | $15^{a}$ | $13^{aa}$ | 35 | 41 | 15 | | 29 | $10^{bb}$ | * | 25 |
| Touch | $6^{a}$ | 8 | 23 | 21 | 13 | | 14 | $4^{bb}$ | | $3^{bb}$ |
| Walking | 2 | – | 2 | 3 | 2 | | – | 2 | | 3 |
| Reaching out | $8^{aa}$ | $3^{aaa}$ | 34 | 38 | 15 | | 29 | $10^{b}$ | * | 25 |
| Carrying | $4^{a}$ | $5^{a}$ | 15 | 18 | 8 | | 20 | 10 | | 20 |
| Working with the arm | $6^{a}$ | $5^{a}$ | 21 | 18 | 8 | | 17 | $6^{b}$ | * | 20 |
| Housework | 9 | $3^{a}$ | 19 | 18 | 2 | ** | 20 | 2 | ** | 20 |
| Handicraft | 2 | 3 | 2 | – | – | | 3 | 2 | * | $15^{b}$ |
| Getting up from bed | $–^{aa}$ | – | 15 | 6 | 2 | | 3 | $4^{b}$ | * | 6 |
| Driving a car | 6 | – | 8 | 3 | – | | – | – | | 3 |
| Writing with the hand | 2 | – | – | – | 4 | | 3 | 6 | | 3 |
| Feeling depressed | 2 | 3 | 4 | 3 | 4 | | 9 | 2 | | 3 |
| Changes in weather | 6 | – | 10 | 5 | 8 | | 9 | 8 | | 8 |

Significant differences between the situation preoperatively and 1 month after surgery ($^{a}P<0.05$, $^{aa}P<0.01$, $^{aaa}P<0.001$), between 1 month and 1 year after surgery ($^{b}P<0.05$, $^{bb}P<0.01$) and between the MRM group ($n=53$, modified radical mastectomy with axillary clearance) and the BCT group ($n=40$, breast resection with axillary clearance, $*P<0.05$, $**P<0.01$).

One year after surgery, sleep was disturbed because of pain in the breast region in 18% and because of pain in the ipsilateral arm in 31% of the patients with chronic pain.

### *Oedema, numbness, strange sensations, muscle weakness, phantom sensations (Table IV) and mobility of the arm*

The incidence of these symptoms (either reported or measured) was not different between patients who had either pN0 or pN1. Neither was there any significant correlation between the incidence of symptoms before and after the operation.

About three out of four patients showed numbness when being examined during the first year after surgery either in the breast region or in the ipsilateral arm. One year after surgery the MRM patients reported significantly ($P<0.01$) more numbness in the operated breast than the BCT patients. However, there were no statistically significant differences between the two groups in the sensory testing. After both types of surgery, measured sensory disturbances were more common ($P<0.001$) than the reported ones. The incidence of measured or reported sensory disturbances did not change during the first post-operative year.

About one-third of the patients had oedema in the ipsilateral upper arm. After both types of surgery, oedema was found to be more common on examination than was reported by the patients. The incidence of the reported oedema in the breast region decreased significantly in the radically operated group between 1 and 6 months after surgery ($P<0.01$), whereas in the conservatively operated group it decreased from 6 months to 1 year after surgery. After MRM, the incidence of oedema in the ipsilateral arm increased significantly from 1 month to 1 year after surgery. Significantly ($P<0.05$) more oedema was measured in the ipsilateral arm in the MRM group than in the BCT group.

About every third patient experienced strange sensations during the first post-operative year either in the breast or in the ipsilateral arm. The incidence of strange sensations in the breast increased significantly from 1 month to 1 year in both surgical groups. The incidence of strange sensations in the ipsilateral arm stayed stable in both groups throughout the year.

The patients in both groups reported significantly less muscle weakness at 6 months than 1 month after surgery. At all time points, the decrease in the gripping force was significantly ($P<0.01$) greater if the dominant side had been operated compared with the non-dominant side.

During the first post-operative year, phantom sensations were reported by one-quarter of the MRM group patients. The incidence of phantom sensations with pain was 23%. The incidence of phantom sensations did not change during the year, and it was not greater in these patients who had experienced pain before the operation, who were younger, or had given birth or lactated.

One month after surgery both pronation and supination were complete in both groups of patients. Abduction and anteversion were reduced by 70–85° in 20% of the patients in the MRM group and 9% in the BCT group (NS). Six months after surgery, the mobility of the arm had completely recovered in all patients after breast resection but was still significantly reduced in 3% of the MRM patients.

### *Anxiety and depression (Table V)*

There were no significant differences in the levels of state and trait anxiety or depression between the types of surgery. The stage of the disease (pTNM) had no correlation with the level of either anxiety or depression. However, the level of trait depression had a significant negative correlation with age at the time of surgery. The marital status of the patient had no

**Table IV** Incidences (% of patients; subjective = reported, objective = measured) of parathesia, oedema, strange sensations, muscle weakness, phantom sensations in the breast region or in the ipsilateral arm after modified radical mastectomy with axillary clearance (MRM, $n=53$) and breast resection with axillary clearance (BCT, $n=40$)

| | *Time after operation* | | | | | |
|---|---|---|---|---|---|---|
| | *1 month* | | *6 months* | | *1 year* | |
| | *Breast* | *Arm* | *Breast* | *Arm* | *Breast* | *Arm* |
| Paraesthesia | | | | | | |
| Mastectomy | | | | | | |
| subjective | 36 ]*** | 60 ]*** | 32 ]*** | 65 | 31 ]*** | 60 ]** |
| objective | 79 | 85 | 71 | 78 | 70 | 81 |
| | * | | | | ** | |
| Resection | | | | | | |
| subjective | 15 ]*** | 62 ]* | 13 ]*** | 63 | 5 ]*** | 65 |
| objective | 66 | 82 | 16 | 79 | 55 | 80 |
| Oedema | | | | | | |
| Mastectomy | | | | | | |
| subjective | 38 | 17 | 8** | 14 ]* | 14* | 20 ]** |
| objective | | 19 | | 38 | | 46** |
| | | | ** | | | * |
| Resection | | | | | | |
| subjective | 30 | 18 | 34 | 13 ]** | 23 | 20 |
| objective | | 25 | | 41 | * | 27 |
| Strange sensations | | | | | | |
| Mastectomy | | | | | | |
| subjective | 26 | 26 | 34 | 14 | 47* | 35 |
| Resection | | | | | | |
| subjective | 23 | 28 | 41 | 28 | 43* | 28 |
| Muscle weakness | | | | | | |
| Mastectomy | | | | | | |
| subjective | | 35 ]* | | 14* | | 16 |
| objective difference | | 13 | | 26 | * | 16 |
| Resection | | | | | | 10 |
| subjective | | 25 | | 14* | | 19 |
| objective difference | | 11 | | 21 | | |
| Phantom sensations | | | | | | |
| Mastectomy | 29 | | 31 | | 25 | |
| subjective | | | | | | |

The incidence of objective paraesthesiae was significantly higher than that of the subjective ones. *$P<0.05$, **$P<0.01$, ***$P<0.001$.

**Table V** The median anxiety scores (possible range 20–80) using Spielberger's STAI (State and Trait Anxiety Inventory) method and the depression scores using an eight-point scale of depression in patients the day before the operation for breast cancer and 1 month, 6 months and 1 year after modified radical mastectomy with axillary clearance (MRM, $n=53$) and breast resection with axillary clearance (BCT, $n=40$)

| | *Trait* | *State Preoperative* | *Post-operative 1 month* | *Post-operative 6 months* | *Post-operative 1 year* |
|---|---|---|---|---|---|
| Anxiety | | | | | |
| Mastectomy | 33.28 (8.74) | 38.98 (9.88) | 35.22 (9.13) | 34.60 (9.56) | 32.34 (9.20)*** |
| | | | | * | |
| Breast resection | 34.35 (7.82) | 41.58 (11.38) | 36.36 (9.96)* | 32.50 (7.19)*** | 32.82 (8.22)*** |
| | | | | * | |
| Total | 33.73 (8.33) | 40.05 (10.54) | 35.75 (9.48)** | 33.69 (8.62)*** | 32.55 (8.75)*** |
| | | | | ** | |
| Depression | | | | | |
| Mastectomy | 2.61 (0.95) | 2.81 (0.98) | 2.68 (0.88) | 2.60 (0.96) | 2.46 (0.86)** |
| | | | | ** | |
| | | | | — * — | |
| Breast resection | 2.64 (0.83) | 3.27 (1.64) | 2.80 (1.08)* | 2.36 (0.56)** | 2.36 (0.93)** |
| | | | | * | |
| Total | 2.62 (0.90) | 3.00 (1.31) | 2.74 (0.98) | 2.49 (0.81)** | 2.42 (0.89)*** |

Significant differences compared with preoperative: *$P<0.05$, **$P<0.01$, ***$P<0.001$.

significant effect on the level of either anxiety or depression. In both groups of patients more than half (MRM 57%, BCT 53%) reported having experienced permanent stressful events within the 6 months preceding breast surgery. The patients who had experienced stressful events connected with work had significantly ($P<0.01$) higher levels of trait anxiety and depression, and they also had significantly ($P<0.05$) more state anxiety and depression both preoperatively and after 1 year from surgery. Difficulties in personal life did not show such a significant impact on the level of either anxiety or depression.

Before surgery, both groups of patients scored significantly higher on the state anxiety scale than 1 year after surgery ($P<0.001$). They were also more depressed before surgery than 1 year after surgery ($P<0.01$). In the BCT group, the levels of both anxiety and depression were already significantly ($P<0.05$) reduced 1 month after surgery. In the MRM patients, the levels of both anxiety and depression only showed a significant reduction compared with presurgical levels 1 year after the operation. There was a significant correlation ($P<0.001$) between trait anxiety and depression and between state anxiety and depression 1 year after surgery.

Both the number of symptoms reported preoperatively ($P<0.05$) in the diseased breast and the number of chronic symptoms in the operated breast region correlated significantly with the level of trait anxiety and trait depression (at 6 months: anxiety $P<0.001$, depression $P<0.05$; at 1 year: anxiety and depression $P<0.01$). One year after surgery, the number of chronic symptoms in the breast region correlated significantly ($P<0.01$) with the level of state anxiety. The number of symptoms reported (only before surgery) in the ipsilateral arm correlated significantly ($P<0.01$) with the level of trait anxiety. The number of symptoms measured in the ipsilateral arm had no significant correlation with any measured levels of anxiety or depression.

### Discussion

This prospective study with 93 women treated for breast cancer shows that 1 year after surgery, most of the women still had treatment-related symptoms in the ipsilateral arm and in the breast area. The incidence of most of the neurological symptoms did not change significantly during the 12 months follow-up. After conservative surgery, there were significantly more symptoms in the ipsilateral arm than in the operated breast.

The role of radiotherapy in brachial plexus neuropathy was recently addressed by the Royal College of Radiologists (Bates, 1995; Maher, 1995). The impact of radiotherapy and other oncological treatments on chronic pain was also analysed in the present patient material. The results will be presented in a future report.

Neither the incidence nor the severity of the preoperative pain correlated with either the size of the tumour or the extent of axillary invasion. This could indicate that the preoperative symptoms are not pathologically based. A more likely explanation is that patients with a knowledge of breast cancer may pay more attention to bodily feelings and link them to their disease. One month after surgery, the incidence of breast pain was similar in both groups. One year after surgery, the incidence of breast pain was higher in the BCT group than in the MRM group. This difference did not reach statistical significance, but it is in agreement with our previous results (Tasmuth *et al.*, 1995), which indicated that more than 1 year after surgery the incidence of breast pain was higher after BCT than MRM. Both Skov *et al.* (1990) and Kroner *et al.* (1992) have reported similar incidences of post-mastectomy pain.

One in ten patients reported pain both in the diseased breast and in the ipsilateral arm before breast surgery. Whereas the incidence of pain in the breast scar was stable throughout the first year, the incidence of pain in the ipsilateral arm tended to decrease. The greatest decrease in the incidence of pain in the ipsilateral arm was from 6 to 12 months in both groups. Ivens *et al.* (1992) have also shown that the pain following damage to the intercostal nerve is relieved with time. The incidence of pain in the ipsilateral arm was 17% in our study, which is the same as that reported by Hladiuk *et al.* (1992). Higher incidences have also been reported (Segerström, 1991; Ivens, 1992; van Dam, 1993). The incidences of pain in the arm were not statistically significant after the two types of surgery which agrees with other studies (Schain, 1983; Stevens, 1995).

One year after surgery, one-third of the patients with pain complained of interrupted sleep due to pain in the region innervated by the intercostobrachial nerve. In a recent study, Stevens *et al.* (1995) reported that half of the patients complained of disturbed sleep due to pain, which also interfered continuously with daily chores. The majority of the patients in our study did not take painkillers. Instead, they employed non-pharmacological approaches including relaxation, massage and exercise.

Within 1 year after surgery, the incidence of most of the factors aggravating post-treatment pain had decreased. Sleeping on the affected (operated) side was the most common factor that aggravated pain. Reaching out, carrying heavy objects, working with the ipsilateral arm and housework were commonly reported as aggravating factors, and they occurred significantly more often after conservative surgery than after radical operation. All these activities are needed in active daily life. Sixty-two per cent of the women in this study were employed, and this is an important fact to be taken into account when assessing a patient's ability to return to work that requires active use of the ipsilateral arm (cleaning, carrying, etc.). Pain evoked by light touch, such as by clothing, is an indication of hyperaesthesia and was found to be present in about 20% of the women 1 month after surgery, decreasing to 3% within a year.

The incidence of paraesthesiae in the region of both the breast and the area innervated by the intercostobrachial nerve did not change during the first post-operative year. Interestingly, the patients reported significantly less numbness than was detected during the neurological examination. This was especially obvious in the breast area, where the patients probably accepted numbness as a natural consequence of the operation, whereas they paid more attention to numbness in the ipsilateral arm.

Various methods have been used in the assessment of oedema in the upper arm. Several groups have compared the circumferences of the ipsi- and contralateral arms (Mondrup *et al.*, 1990; Olsen *et al.*, 1990; Keramopoulus *et al.*, 1993). We, like Gerber *et al.* (1992), compared post-operatively the circumference of the ipsilateral arm with the preoperatively measured values. In all these studies, the criterion for oedema has been an increase of at least 2 cm in either the arm or the forearm measurement. Significantly more oedema was detected than was reported spontaneously by the patients. This has also been reported by Kissin *et al.* (1986). Post-treatment oedema in the arm was significantly more common in the group treated with radical surgery than after conservative surgery. This disagrees with what Gerber *et al.* (1992) have reported. The incidence of oedema in the arm increased over time, which agrees with other studies (Hladiuk, 1992). The incidence of objective oedema as defined above was 27–46% in our study, which agrees well with Kissin *et al.* (1986), who reported an incidence of 38% in patients who had axillary lymph node clearance and radiation therapy.

In order to assess changes in muscle strength, we used a method in which we compared the gripping force of the operated hand before and after treatment. There were no significant differences in the gripping force after radical or conserving surgery. Muscle weakness was reported most commonly 1 month after surgery, after which the incidence decreased. Interestingly, the number of patients with a decrease in the gripping force of at least 20% compared with the preoperatively measured value was significantly smaller if the dominant side had been operated. This could indicate that active use of the dominant hand improved the recovery of muscle strength. Previous studies which have used the same type of dynamometer have shown that the grip strength in the ipsilateral side compared with the contralateral side has been reduced by 12–18% after breast surgery (Hladiuk *et al.*, 1992).

The incidence of phantom sensations in the present study was 25%. This is similar to that reported by Kroner *et al.* (1992), but markedly lower than that reported by Jamison *et al.* (1979), Downing *et al.* (1984) and Karydas *et al.* (1986). The incidence of phantom sensation with pain has been reported to be about 20% (Weinstein *et al.*, 1970; Kroner *et al.*, 1989); in our study it was 23%. The incidence of phantom sensations did not change significantly during the first year. Kroner *et al.* (1992) have reported that phantom sensations can last unchanged for more than 6 years. A few studies have indicated that young patients are more prone to developing phantom sensations (Weinstein *et al.*, 1970; Jamison *et al.*, 1979; Downing *et al.*, 1984; Staps *et al.*, 1985). This assumption was not supported by the results of our study, which is in accordance with the results of Kroner *et al.* (1992). It has been suggested that pain preceding the amputation would predispose to phantom pain (Kroner *et al.*, 1989). In our study, however, preoperative pain had no effect on the incidence of phantom sensations.

The mobility of the arm was preserved better in our patients than reported previously (Hladiuk *et al.*, 1992; Thompson *et al.*, 1995). This could be a result of the active physiotherapy that was included in the rehabilitation programme.

We chose the method developed by Spielberger to measure anxiety and the method developed by Kanerva to measure depression (Poikolainen *et al.*, 1995) because these methods were recently evaluated in healthy Finnish-speaking women. A significant correlation between state depression ($n=927$, $r=0.61$) and trait depression ($n=927$, $r=0.67$), measured using this method and the Beck Depression Inventory, has been shown (AR Aro, unpublished data). The State and Trait Anxiety Inventory has been used previously by Thomas *et al.* (1995) and Liu *et al.* (1994) to study the correlations between anxiety and pain. Previously, Richter *et al.* (1991) and Kavoussi *et al.* (1993) have applied 'trait depression' to the MMPI (The Minnesota Multiphasic Personality Inventory) scales. 'State depression' has been assessed by the Beck Depression Inventory (Richter *et al.*, 1991) and the Hamilton Depression Rating Scale (Kavoussi *et al.*, 1993).

In our study, both the trait anxiety and depression were somewhat lower in our patients than in the healthy 50-year-old Finnish-speaking women examined in 1992–93 by Aro (1996). The levels of state anxiety and depression in the study by Aro (1996) are in accordance with our results at 6 months after surgery. The patients were significantly more depressed and anxious before surgery than after 1 year. This agrees with the results of Goldberg *et al.* (1992). In our study, we were also unable to show any difference between the two surgical groups, and neither anxiety nor depression correlated with the state of the disease. Previously, Fallowfield *et al.* (1990) and Goldberg *et al.* (1992) have also reported no difference in psychological distress between patients who have had either radical or conservative therapy. These facts could indicate that the majority of psychological distress stems from the diagnosis of cancer rather than from the type of primary treatment (Fallowfield *et al.*, 1987; Schain *et al.*, 1983). However, both state anxiety and depression had significantly decreased 1 month after surgery in the BCT group, whereas no significant differences were obvious in the MRM group at that time. Interestingly, a similar tendency for more rapid decreases in the profile of mood states in BCT patients than in MRM patients has been reported by Ganz *et al.* (1992). This could indicate that psychic recovery takes longer after more extensive surgery.

There seems to be an association between the levels of anxiety and depression and preoperative stressful factors at work, whereas stressful events in personal life seem less important. This is significant as about 62% of the patients were still working. The great impact of work-related stressful factors may be explained by the fact that the study was performed during the deepest period of the economic recession in Finland, where the rate of unemployment increased 4-fold in a few years. Maunsell *et al.* (1992) have also shown that stressful life events before diagnosis appear to be a strong indicator of the risk of psychological distress. History of depression is another risk factor, whereas age, education and marital status seem to have little or no association with levels of psychological distress.

In our study, the level of anxiety and depression correlated significantly with the prevalence of symptoms in the breast region. This relationship between chronic symptoms and mood has previously been suggested by Spiegel *et al.* (1988) and by Tobin *et al.* (1993). The latter study showed that patients with arm swelling experienced greater psychiatric morbidity. This could indicate that chronic symptoms are a constant reminder of the disease and thus nurture psychic distress.

This study was performed in a university hospital department that is a centre for the treatment of breast cancer. Thus, the results of this report may not be wholly generalisable. The patients in this study had no signs of the spread of the disease. The symptoms following more aggressive treatments can be assumed to be both more frequent and more severe.

This study shows that the incidence of chronic post-treatment pain and other symptoms is considerable and may affect functions that are important to women in their activities both at work and at home. Chronic post-treatment symptoms should be taken into account when informing patients about treatment possibilities. After treatment patients should be asked about these symptoms as chronic pain, for example, can be effectively treated with amitriptyline (Kalso *et al*., 1996). Ongoing studies are needed to determine whether more careful surgery and radiotherapy, and planning of the oncological treatments can diminish the incidence of these symptoms.

**Acknowledgements**

This study was financially supported by the Academy of Finland (TT and EK) and the Centre for International Mobility (TT).

### References

ARO AR. (1996). *Psychosocial Factors associated with Participation in Mammography Screening*, Academic Dissertation (in Finnish with a summary in English). University of Turku, Publications of the National Public Health Institute, A2.

ASPEGREN K, HOLMBERG L AND ADAMI OH. (1988). Standardization of the surgical technique in breast-conserving treatment of mammary cancer. *Br. J. Surg.*, **75,** 807–810.

BATES TD AND EVANS RGB. (1995). *Brachial Plexus Neuropathy following Radiotherapy for Breast Carcinoma*. Royal College of Radiologists: London.

DOWNING R AND WINDSOR CWO. (1984). Disturbance of sensation after mastectomy. *Br. Med. J.*, **288,** 1650.

FALLOWFIELD LJ, BAUM M AND MAGUIRE GP. (1987). Addressing the psychological needs of the conservatively treated breast cancer patient: discussion paper. *J. Roy. Soc. Med.*, **80,** 696–700.

FALLOWFIELD LJ, HALL A, MAGUIRE GP AND BAUM M. (1990). Psychological outcomes of different treatment policies in women with early breast cancer outside a clinical trial. *Br. Med. J.*, **301,** 575–580.

FISHER B, ANDERSON S, REDMOND CK, WOLMARK N, WICKERHAM DL AND CRONIN WM. (1995). Reanalysis and results after 12 years of follow-up in a randomized clinical trial comparing total mastectomy with lumpectomy with or without irradiation in the treatment of breast cancer. *N. Eng. J. Med.*, **333,** 1456–1461.

GANZ PA, SCHAG AC, LEE JJ, POLINSKY ML AND TAN S-J. (1992). Breast conservation versus mastectomy: is there a difference in psychological adjustment or quality of life in the year after surgery. *Cancer*, **69,** 1729–1738.

GERBER L, LAMPERT M, WOOD C, DUNCAN M, D'ANGELO T, SCHAIN W, MCDONALD H, DANFORTH D, FINDLAY P, GLATSTEIN E, LIPPMAN ME, STEINBERG SM, GORRELL C, LICHTER A AND DEMOSS E. (1992). Comparison of pain, motion, and edema after modified radical mastectomy vs. local excision with axillary dissection and radiation. *Breast Cancer Res. Treat.*, **21,** 139–145.

GOLDBERG JA, SCOTT RN, DAVIDSON PM, MURRAY GD, STALLARD S, GEORGE WD AND MAGUIRE GP. (1992). Psychological morbidity in the first year after breast surgery. *Eur. J. Surg. Oncol.*, **18,** 327–331.

HLADIUK M, HUCHCROFF S, TEMPLE W AND SCHNURR BE. (1992). Arm function after axillary dissection for breast cancer: a pilot study to provide parameter estimates. *J. Surg. Oncol.*, **50,** 47–52.

HUSKISSON EC. (1974). Measurement of pain. *Lancet*, **2,** 1127–1131.

IVENS D, HOE AL, PODD TJ, HAMILTON CR, TAYLOR I AND ROYLE GT. (1992). Assessment of morbidity from complete axillary dissection. *Br. J. Cancer*, **66,** 136–138.

JAMISON K, WELLISCH DK AND KATZ RL. (1979). Phantom breast syndrome. *Arch. Surg.*, **114,** 93–95.

KALSO E, TASMUTH T AND NEUVONEN PJ. (1996). Amitriptyline effectively relieves neuropathic pain following treatment of breast cancer. *Pain*, **64,** 293–302.

KARYDAS I, FENTIMAN IS, HABIB F AND HAYWARD JL. (1986). Sensory changes after treatment of operable breast cancer. *Breast Cancer Res. Treat.*, **8,** 55–59.

KAVOUSSI RJ, COCCARO EF, KLAR H, LESSER J AND SIEVER LJ. (1993). The TRH-stimulation test in DSM-III personality disorder. *Biol. Psychiatry*, **34,** 234–239.

KERAMOPOULOS A, TSIONOU C, MINARETZIS D, MICHALAS S AND ARAVANTINOS D. (1993). Arm morbidity following treatment of breast cancer with total axillary dissection: a multivariate approach. *Oncology*, **50,** 445–449.

KISSIN MW, DELLA REVERE GQ, EASTON D AND WESTBURY G. (1986). Risk of lymphoedema following the treatment of breast cancer. *Br. J. Surg.*, **73,** 580–584.

KRØNER K, KREBS B, SKOV J AND JØRGENSEN HS. (1989). Immediate and long-term phantom breast syndrome after mastectomy: incidence, clinical characteristics and relationship to pre-mastectomy breast pain. *Pain*, **36,** 327–334.

KRØNER K, KNUDSEN UB, LUNDBY L AND HVID H. (1992). Long-term phantom breast syndrome after mastectomy. *Clin. J. Pain.*, **8,** 346–350.

KUUSK U, BASCO V AND REBBECK P. (1992). Comparison of partial and modified radical mastectomy in the community setting – '10 years later'. *C.J.S.*, **35,** 383–387.

LIU R, BARRY JE AND WEINMAN J. (1994). Effects of background stress and anxiety on postoperative recovery. *Anesthesiology*, **49,** 382–386.

MAHER EJ. (1995). *Management of Adverse Effects following Breast Radiotherapy*. Royal College of Radiologists: London.

MAUNSELL E, BRISSON J AND DESCHÊNES L. (1992). Psychological distress after initial treatment of breast cancer. *Cancer*, **70,** 120–125.

MONDRUP K, OLSEN NK, PFEIFFER P AND ROSE C. (1990). Clinical and electrodiagnostic findings in breast cancer patients with radiation-induced brachial plexus neuropathy. *Acta Neurol. Scand.*, **81,** 153–158.

OLSEN NK, PFEIFFER P, MONDRUP K AND ROSE C. (1990). Radiation-induced brachial plexus neuropathy in breast cancer patients. *Acta Oncol.*, **29,** 885–890.

OLSEN NK, PFEIFFER P, JOHANSSEN L, SCHRØDER H AND ROSE C. (1993). Radiation-induced brachial plexopathy: neurological follow-up in 161 recurrence-free breast cancer patients. *Int. J. Radiat. Oncol. Biol. Phys.*, **26,** 43–49.

OMNE-PONTÉN M, HOLMBERG L, BURNS T, ADAMI HO AND BERGSTRÖM R. (1992). Determinants of the psycho-social outcome after operation for breast cancer. Results of a prospective comparative interview study following mastectomy and breast conservation. *Eur. J. Cancer*, **28A,** 1062–1067.

POIKOLAINEN K, KANERVA R AND LÖNNQVIST J. (1995). Life events and other risk factors for somatic symptoms in adolescence. *Pediatrics*, **96,** 59–63.

RAYTER Z, GAZET J-C, FORD HT, EASON DF AND COOMBES RC. (1990). Comparison of conservative surgery and radiotherapy with mastectomy in the treatment of early breast cancer. *Eur. J. Surg. Oncol.*, **16,** 486–492.

READ PE, ASH DV, THOROGOOD J AND BENSON EA. (1987). Short term morbidity and cosmesis following lumpectomy and radical radiotherapy for operable breast cancer. *Clin. Radiol.*, **38,** 371–373.

RICHTER J, EISEMANN M AND RICHTER G. (1991). Perceived parental rearing and state versus trait aspects of adult depression. *Psychopathology*, **24,** 25–30.

SCHAIN W, EDWARDS BK, GORRELL CR, DE MOSS EV, LIPPMAN ME, GERBER LH AND LICHTER AS. (1983). Psychosocial and physical outcomes of primary breast cancer therapy: mastectomy vs excisional biopsy and irradiation. *Breast Cancer Res. Treat.*, **3,** 377–382.

SEGERSTRÖM K, BJERLE P AND NYSTRÖM Å. (1991). Importance of time in assessing arm and hand function after treatment of breast cancer. *Scand. J. Plast. Reconstr. Hand Surg.*, **25,** 241–244.

SKOV J, KRØNER K, KREBS B, HVID HM AND JØRGENSEN HS. (1990). Cicatricielle smerter og dysæstesier efter ablatio mammae. Ugeskr. Laeger, **152,** 3081–3084.

SPIEGEL D AND SANDS SH. (1988). Pain management in the cancer patient. *J. Psychosoc. Oncol.*, **6,** 205–216.

SPIELBERGER CD. (1975). *The Measurement of State and Trait Anxiety: Conceptual and Methodological Issues*. Raven Press: New York.

STAPS T, HOOGENHOUT J AND WOBBES T. (1985). Phantom breast sensations following mastectomy. *Cancer*, **56,** 2898–2901.

STEVENS PE, DIBBLE SL AND MIASKOWSKI C. (1995). Prevalence, characteristics, and impact of postmastectomy pain syndrome: an investigation of women's experiences. *Pain*, **61,** 61–68.

TASMUTH T, VON SMITTEN K, HIETANEN P, KATAJA M AND KALSO E. (1995). Pain and other symptoms after different treatment modalities of breast cancer. *Ann. Oncol.*, **6,** 453–459.

THOMAS V, HEALTH M, ROSE D AND FLORY P. (1995). Psychological characteristics and the effectiveness of patient-controlled analgesia. *Br. J. Anaesth.*, **74,** 271–276.

THOMPSON AM, AIR M, JACK WJL, KERR GR, RODGER A AND CHETTY U. (1995). Arm morbidity after breast conservation and axillary therapy. *The Breast*, **4,** 273–276.

TOBIN MB, PSYCH MRC, LACEY HJ, PSYCH FRC, MEYER L AND MORTIMER PS. (1993). The psychological morbidity of breast cancer-related arm swelling: Psychological morbidity of lymphoedema. *Cancer*, **72,** 3248–3252.

VAN DAM MS, HENNIPMAN A, DE KRUIF JT, VAN DER TWEEL I AND DE GRAAF PW. (1993). Complicaties na okselkliertoilet wegens mammacarcinoom. *Ned. Tijdschr. Geneeskd.*, **137,** 2395–2398.

WEINSTEIN S, VETTER RJ AND SERSEN EA. (1970). Phantoms following breast amputation. *Neuropsychology*, **8,** 185–197.